# Co-occurrence of Pain Symptoms and Somatosensory Sensitivity in Burning Mouth Syndrome: A Systematic Review

**DOI:** 10.1371/journal.pone.0163449

**Published:** 2016-09-22

**Authors:** Xavier Moisset, Valentina Calbacho, Pilar Torres, Christelle Gremeau-Richard, Radhouane Dallel

**Affiliations:** 1 Clermont Université, Université d’Auvergne, Neuro-Dol, Clermont-Ferrand & Inserm U1107, Clermont-Ferrand, France; 2 CHU Clermont-Ferrand, Service d’Odontologie, Clermont-Ferrand, France; 3 CHU Clermont-Ferrand, Service de Neurologie, Clermont-Ferrand, France; 4 Facultad De Odontología, Universidad San Sebastián, Santiago, Chile; Duke University, UNITED STATES

## Abstract

**Background:**

Burning mouth syndrome (BMS) is a chronic and spontaneous oral pain with burning quality in the tongue or other oral mucosa without any identifiable oral lesion or laboratory finding. Pathogenesis and etiology of BMS are still unknown. However, BMS has been associated with other chronic pain syndromes including other idiopathic orofacial pain, the dynias group and the family of central sensitivity syndromes. This would imply that BMS shares common mechanisms with other cephalic and/or extracephalic chronic pains. The primary aim of this systematic review was to determine whether BMS is actually associated with other pain syndromes, and to analyze cephalic and extracephalic somatosensory sensitivity in these patients.

**Methods:**

This report followed the PRISMA Statement. An electronic search was performed until January 2015 in PubMed, Cochrane library, Wiley and ScienceDirect. Searched terms included “burning mouth syndrome OR stomatodynia OR glossodynia OR burning tongue OR oral burning”. Studies were selected according to predefined inclusion criteria (report of an association between BMS and other pain(s) symptoms or of cutaneous cephalic and/or extracephalic quantitative sensory testing in BMS patients), and a descriptive analysis conducted.

**Results:**

The search retrieved 1512 reports. Out of these, twelve articles met criteria for co-occurring pain symptoms and nine studies for quantitative sensory testing (QST) in BMS patients. The analysis reveals that in BMS patients co-occurring pain symptoms are rare, assessed by only 0.8% (12 of 1512) of the retrieved studies. BMS was associated with headaches, TMD, atypical facial pain, trigeminal neuralgia, post-herpetic facial pain, back pain, fibromyalgia, joint pain, abdominal pain, rectal pain or vulvodynia. However, the prevalence of pain symptoms in BMS patients is not different from that in the age-matched general population. QST studies reveal no or inconsistent evidence of abnormal cutaneous cephalic and extracephalic somatosensory sensitivity.

**Conclusions:**

There is no evidence for a high rate of other pain symptoms or somatosensory impairments co-occurring with BMS. These results thus suggest that BMS rather depends on specific mechanisms, likely at the trigeminal level. Nevertheless, more thoroughly conducted research is required to draw definitive conclusion.

## Introduction

Burning mouth syndrome (BMS) is a chronic and spontaneous non remitting oral pain with burning quality in the tongue or other oral mucosa without any identifiable local lesion or laboratory finding [1,2]. BMS prevalence increases with age, the highest being in 60–69 years-old women [1–4]. The burning pain can be localized to entire oral mucosa, most often the tongue, the lip and the palate. The burning sensation is often accompanied by oral dysesthesia, decrease or impaired taste, along with a feeling of abnormal saliva often identified as xerostomia [1,2].

The pathogenesis and etiology of BMS are still unknown. This disease was initially classified as a psychalgic pain [5–7]. But recent evidence suggest that it is rather a peripheral and/or central neuropathic disorder [3,4]. Nevertheless, the cause of such neuropathic changes is still unknown. Of note, combined dysregulation of adrenal, gonadal and neuroactive steroids [8] or dysfunction of gustatory and somatic afferents [9,10] have been hypothesized.

BMS might also be only a pain symptom, which would then be associated with other cephalic and/or extra-cephalic pain symptoms. For instance, it was proposed that BMS and other chronic orofacial pains, such as atypical odontalgia, atypical facial pain and arthromyalgia, are parts of a single disease: they would have the same underlying pathophysiological mechanisms but different tissue expressions [1]. BMS might also be related to dynias, a group of chronic, focal pain syndromes of the orocervical and urogenital regions, including carotidynia, vulvodynia, orchidynia, prostatodynia, coccygodynia and proctodynia [11]. Finally, recent studies suggest that any orofacial pain without organic cause belongs to central sensitivity syndromes that are produced by central sensitization [12–15]. Consistent with the last hypothesis, BMS can coexist with other chronic extracephalic pain conditions such as fibromyalgia and visceral pain [16]. It is therefore possible that BMS shares common mechanisms with other chronic cephalic and/or extracephalic pain syndromes. We reasoned that, if this is true, BMS patients should exhibit other pain symptoms as well as abnormal cephalic and/or extracephalic somatosensory sensitivity. Thus, the aims of this systematic review were to assess (i) co-occurring pain symptoms, and (ii) changes in cephalic and extra-cephalic somotosensory sensitivity, in patients with BMS.

## Methods

### Study selection

The present study followed the PRISMA statement guidelines [17] (S1 Fig). A computerized literature search for articles published until January 15^th^, 2015, was conducted in the databases: PUBMED, Cochrane library, Wiley and ScienceDirect. We used Cited Reference Search (Web of Science) to find articles in identified publications (February 5^th^, 2015). In addition, the reference lists of all retrieved articles were further examined to identify additional relevant articles that were not detected by the initial search. Our search was limited to human studies published in English or French. The following key words were used: “burning mouth syndrome OR stomatodynia OR glossodynia OR burning tongue OR oral burning”.

Studies to be included in this review had to match predetermined criteria according to the PICOS (participants, interventions, comparators, outcomes, and study design) approach. Criteria for inclusion and exclusion are specified in Table 1. To be eligible, studies needed to include BMS patients over 15 years-old and either examine the occurrence of other pain symptoms or quantify sensory function (using quantitative sensory testing, QST). We included studies looking for a link between BMS and other types of pain. For QST, we restricted our selection to studies focusing on cutaneous thermal or mechanical sensitivity in cephalic and extracephalic regions. On the other hand, studies focusing solely on psychological or potentially allergic factors and case reports were excluded (Table 1).

**Table 1:** PICOS criteria for inclusion and exclusion of studies.

Parameter	Inclusion criteria	Exclusion criteria
Patients	• Patients ≥ 15 years. • Patients referred as burning mouth syndrome	Patients under 15 years of age
Intervention	Not applicable	
Comparator	Studies with control/comparison groups	
Outcome	1. Presence of other chronic cephalic and/or extracephalic pain 2. Cutaneous cephalic and/or extracephalic quantitative sensory testing	Studies focusing solely on psychological or potentially allergic factors. Studies on animals
Study Design	Randomized controlled trials, retrospective, prospective, or concurrent cohort studies, cross sectional studies	• Reviews, expert opinion, comments, letter to editor, case reports, conference reports. • Studies published in any other language than English or French

PICOS = patients, intervention, comparator, outcomes, study design.

Two investigators independently conducted the search. Articles that met the selection criteria as well as those with imprecise abstracts according to these criteria were considered for full-text analysis.

### Data extraction

Two investigators independently extracted and included all relevant data in tables. The following study characteristics and outcome data of interest were extracted: number of participants, participants’ characteristics (age, sex, diagnosis, duration of symptoms), BMS diagnostic criteria, prevalence of co-occurring pain conditions, QST measures, thermode size (for thermal stimulation) and quantitative values for histological parameters. Discrepancies between investigators on study selection and data extraction were resolved by discussion and consensus.

### Risk of bias assessment

As proposed in the recent systematic review on neuropathic pain prevalence by van Hecke and coworkers [18], all articles that fully met the inclusion criteria were critically appraised using the STROBE checklist (Strengthening the Reporting of OBservational studies in Epidemiology) [19]. This is a structured, standardized checklist consisting of 22 items, each relating to the different sections in an article (ie, title, abstract, introduction, methods, results, discussion, and funding), its main purpose being to improve the transparency of reporting in epidemiological observational research.

Quality factors based on key items of the STROBE quality checklist instrument were combined to form a modified checklist consisting of a total of 30 items with a simple point system. Each article was then given a total score out of 30. The quality scores calculated for each article gave comparisons of the relative quality of included studies, a higher score indicating higher quality. A relatively low score did not necessarily imply poor-quality research, as the score was a guide to the quality of reporting according to a specific (STROBE) checklist.

### Data Analysis

For co-occurring pain symptoms in BMS patients, both absolute and normalized numbers of patients were extracted. Pains were classified into two main groups according to their location: cephalic (temporomandibular disorder (TMD), headaches, atypical facial pain (AFP), trigeminal neuralgia) and extracephalic (abdominal, genital, back, widespread musculoskeletal (fibromyalgia) and joint pain). QST measurements were classified into cephalic skin or upper limb skin measurements. When available, means and standard deviations of temperature thresholds (in Celsius degree; °C) were noted. For analysis, thresholds in BMS patients were compared with those in controls.

## Results

### Selected studies

**Fig 1:**
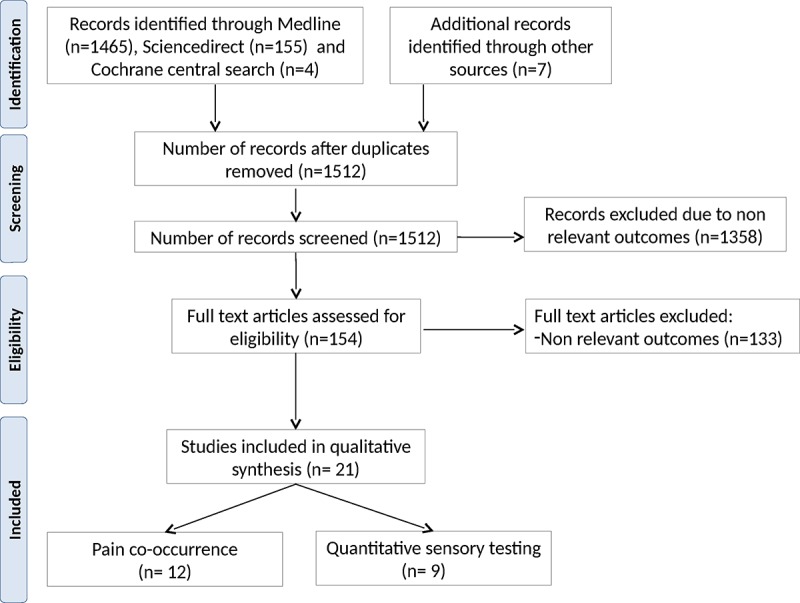
PRISMA flow diagram of process of identification and screening of articles for inclusion in this review.

According to the selected criteria, we identified 1512 reports (Fig 1). Out of these, 12 articles including 633 patients met all criteria for examining co-occurring pain symptoms [16,20–30] and 9 studies, including 384 participants for quantitative sensory testing [31–39].

### Pain co-occurrence in patients with BMS

Studies looking at co-occurring pain symptoms were only 0.8% (12 of 1512) of the retrieved reports. The mean age of the 633 included patients was 60.9 years-old; women (85%) were more often seen than men. In two studies, the main inclusion criterion was oral burning sensation, without any other criterion for BMS [25,29]. In the remaining ones, patients were probably BMS but the 2013 IHS criteria were not applied [40]. Of note, only one study was designed to specifically address somatic comorbidities [16]. Therefore, there might be a risk of detection bias for all others.

#### Cephalic pain symptoms

Headache was the most frequent cephalic pain symptom associated with BMS: it occurred in altogether 84 patients [16,23–25,30], that is, 13.3% of the total number of included patients (12 studies) and 22.8% of patients included in these very 5 studies (Table 2). TMD was reported in 46 patients [20,21,29], atypical facial pain in 7 patients [25,30], trigeminal neuralgia in 2 patients [27] and post-herpetic facial pain in a single patient [30]. In one study, the prevalence of TMD was found to be the same in BMS patients and controls [28].

**Table 2:** Summary of studies reporting co-occurrent pain symptoms in burning mouth syndrome (BMS) patients.

Study	Type of study	Sample size(% female)	Mean age in years (SD/range)	Pain duration	Inclusion criteria	Co-occurrent pain	Quality assessment score (max 30)
Bergdahl *et al*, 1994 [20]	Consecutive	17 (ND)	53 (34–79)	ND	+	6 TMD, several headache (ND)	16
Corsalini *et al*., 2013 [21]	Consecutive	44 (86)	67 (45–89)	ND	+	29 TMD	20
Forssell *et al*., 2002 [22]	Consecutive	52 (88)	60* (30–82)	> 4 m	+	10 back pain, joint pain or fibromyalgia	20
Forssell *et al*., 2012 [23]	Consecutive	52 (100)	63 (33–82)	≥ 3 m	+	6 headache, 7 joint pain, 4 fibromyalgia	19
Grushka *et al*., 1987 [24]	Consecutive	72 (85)	? (33–84)	> 3 m	+	38 headache	17
Hakeberg *et al*., 1997 [25]	Epidemiology survey	47 (100)	68 (38–84)	≥ 6 m	-	19 headache, 5 AFP	22
Lamey *et al*., 2005 [26]	Questionnary	84 (88)	65 (25–97)	<12–> 132 m	+	37 back pain	25
Mignogna *et al*., 2011 [16]	Prospective	124 (73)	57 (12)	≥ 6 m	+	23 back pain, 21 abdominal pain, 20 headaches, 8 joint pain, 7 muscle pain, 4 vulvodynia, 3 rectal pain	26
Nasri *et al*., 2007 [27]	Consecutive	66 (85)	62 (35–83)	1–360 m	+	2 trigeminal neuralgia, 4 fibromyalgia	20
Netto *et al*., 2011 [28]	Case-control	32 (72)	61 (27–87)	ND	+	No difference for TMD	27
Thorstensson and Hugoson, 1996 [29]	Epidemiology survey	18 (89)	51 (20–70)	ND	-	11 TMD (association between burning sensation and TMD)	24
Woda *et al*., 1998 [30]	Consecutive	25 (84)	62 (39–83)	≥ 4 m	+	9 back pain, 3 vulvodynia, 2 AFP, 1 diffuse myalgia, 1 TTH, 1 post-herpetic pain	15

AFP, Atypical facial pain; TMD, Temporomandibular disorder; TTH, Tension-type headache; ND, Not determined

*Median

-, poor definition of BMS

+, correct enough definition of BMS.

#### Extracephalic pain symptoms

Back pain was noted for 69 individuals [16,26,30], i.e. in 29.6% of patients included in these very 3 studies (Table 2). Fibromyalgia or the description of muscular pain was reported in 16 patients [16,23,27,30], joint pain in 15 patients [16,23], abdominal pain in 21 patients–i.e. in 16.9% of the study sample in [16]–rectal pain in 3 patients [16], and vulvodynia in 7 patients [16,30].

### Somatosensory sensitivity

Nine studies performed QST to detect somatosensory abnormalities (Table 3). Sample sizes ranged from 8 to 41, with 384 participants overall (180 patients and 204 normal subjects). Studies included significantly (χ2: 16.84; P< 0.0001) more women in the patient group (92%) than in the control group (68%). The mean age of participants was similar in both groups (patients: 59.7 ± 3.0 years, range 49–67 years; controls: 60.2 ± 3.5 years, range 49–69 years, mean ± SEM).

**Table 3:** Somatosensory sensitivity in patients with burning mouth syndrome (BMS).

Study	• Sample size (% female) • Mean age in years (SD/range)		Pain duration	Thermode size (mm)	Cephalic skin					Extra-cephalic skin	Quality assessment score (max 30)
	Patients	Controls			WDT	HPT	CDT	CPT	MPT	HPT	
**Albuquerque et al., 2006 [31]**	• 8 (100) • 49 (41–71)	• 8 (100) • 50 (12)	> 24 m	30x30		NS					28
**Grémeau-Richard et al., 2010 [32]**	• 20 (100) • 65 (8)	• 20 (100) • 61 (7)	≥ 4 m	25x50		NS				hyper	27
**Grushka et al., 1987 [33]**	• 40 (?) • ?	• 23 (?) • ?	≥ 3 m	7x7	NS	NS			NS		17
**Ito et al., 2002 [34]**	• 20 (100) • 52 (43–64)	• 20 (100) • 49 (35–59)	> 36 m	?						NS	18
**Kaplan et al., 2011 [35]**	• 26 (?) • > 50	• 24 (?) • > 50	ND	20x20	NS	NS	NS	NS			21
**Kutscher and Chilton, 1952 [36]**	• 15 (73) • ?	• 15 (73) • ?	ND	NA (radiant heat)						NS	7
**de Siqueira et al., 2013 [37]**	• 8 (100) • 67 (37–88)	• 41 (46) • 64 (20)	≥ 6 m	9x9	NS				NS		26
**Siviero et al., 2011 [38]**	• 20 (80) • 61 (12)	• 30 (33) • 69 (10)	> 36 m	?	hypo		NS		• hypo V2 • NS V3		25
**Svensson et al., 1993 [39]**	• 23 (96) • 64 (50–87)	• 23 (91) • 68 (46–81)	ND	NA (Laser)	hypo	hypo				hypo	21

CDT, cold detection threshold; CPT, cold pain threshold; HPT, heat pain threshold; hypo, hyposensitivity; hyper, hypersensitivity; M: mechanical; NA, Not applicable; NS, no significant difference compared to the control group;?, missing information.

QST was performed in both cephalic and extra-cephalic cutaneous areas. Various cephalic (lip, chin, masseter region, cheek, nose wing,…) and extra-cephalic sites (finger, dorsum of the hand, anterior tibia, wrist) were stimulated [31–39]. Cephalic and extra-cephalic QST led to conflicting results (Table 3). The cephalic cutaneous somatosensory sensitivity was investigated in seven studies. Two [38,39] of the five studies testing warm sensitivity reported increased WDT in BMS patients compared with controls, and the remaining three [33,35,37] no difference. Four of the five studies [31–33,35] evaluating HPT found no difference, while the last one [39] described a higher HPT in BMS patients compared with controls. The CDT of BMS patients was reported to be similar to that of controls [35,38]. Grushka and coworkers [33,41] were the first to study the cutaneous mechanical sensitivity and did not find any change in mechanical thresholds in the mandibular area in BMS patients compared with controls [33,41][33,41]. Similar results were obtained by another group [37,42]. However, one study found mechanical hypoalgesia in the maxillary area but no change in the mandibular one [38].

Finally, four studies also investigated extra-cephalic cutaneous somatosensory sensitivity in BMS patients. HPT on the upper limb of BMS patients was reported to be higher [39], lower [32] or similar to control subjects [34,36].

## Discussion

The present work is the first systematic literature review aimed to determine whether BMS is actually associated with other pain syndromes, and to analyze cephalic and extracephalic somatosensory sensitivity in BMS patients. We found that the co-occurrence of BMS with other pain symptoms is assessed in less than 1% of the retrieved studies and there is no or inconsistent evidence of abnormal cutaneous cephalic and extracephalic somatosensory sensitivity in BMS patients.

### Co-occurrent pain symptoms in BMS patients

The selected studies report that BMS is associated with headaches, TMD, atypical facial pain, trigeminal neuralgia, post-herpetic facial pain, back pain, fibromyalgia, joint pain, abdominal pain, rectal pain or vulvodynia. However, the prevalence of pain symptoms in BMS patients is not different from that in the age-matched general population [43–50] (Table 4). A single study was designed to specifically evaluate the prevalence of extracephalic pain in BMS patients: it concludes that these patients present several additional unexplained extraoral comorbidities [16]. Unfortunately, control subjects in this study appear to be rather healthy: only 4% exhibited headache and none back pain. Thus, they may not be representative of the general population since headache and back pain are reported in 46% and 29% of the (matched) general population, respectively [48,50]. Therefore, there is an absence of any study that properly assessed overlapping pain conditions in BMS and more rigorously conducted research is required to allow definitive conclusion.

**Table 4:** Prevalence of co-occurrent pain symptoms in burning mouth syndrome (BMS) patients’ samples and in the general population.

Pain syndromes	Prevalence of the pain symptom in the study sample	Prevalence of the pain symptom in the general population of equivalent age
Temporomandibular disorders	12 to 66% [20,21,29,30]	44 to 50% [44,47]
Headaches	4 to 40% [16,23,25,30,41]	46% with active headache [50]
Abdominal pain	17% [16]	IBS in 6–18% [43]
Genital pain	2 to 12% [16,30]	7 to 8% [45,49]
Back pain	19 to 44% [16,26,30]	29% [48]
Widespread musculoskeletal pain (fibromyalgia)	4 to 8% [16,23,27,30]	2 to 8% [46]

### Cephalic and extra-cephalic cutaneous sensitivity

Quantitative sensory testing (QST) is a psychophysical method widely used to quantify somatosensory function in healthy subjects and patients [51]. QST has been used for decades in research settings, particularly for diagnosing, assessing, and monitoring sensory neuropathies and pain disorders [51]. In BMS patients, nine studies have used QST to evaluate thermal and mechanical cephalic and extracephalic somatosensation. All [31–33,35] but one study [39] reported normal cephalic HPT in BMS patients. BMS patients showed either warm hypoesthesia [38,39] or normal warm sensation [33,35,37]. According to cold perception, cephalic CDT was found normal in two studies [35,38].

The single study that assessed extra-cephalic CPT in BMS patients found no change in cold pain [35]. Extra-cephalic HPTs were reported to be higher [39], lower [32] or similar in BMS patients compared with healthy controls [34,36]. Thus, altogether, it is not possible to definitely conclude that BMS is associated with abnormal cephalic and extra-cephalic somatosensory sensitivity.

### Limitations of reviewed studies

The selected studies were performed between 1952 and 2014. No study has used the latest IHS criteria [40]. Moreover, the earliest diagnostic criteria being published in 1994 (see Table 5), studies conducted before this date must be cautiously interpreted when precise inclusion criteria are not specified. Finally, authors using IASP criteria can include heterogeneous patients [52], some of them presenting burning sensations that do not fulfill current BMS criteria.

**Table 5:** Evolution of burning mouth syndrome (BMS) diagnostic criteria.

1988	ICHD 1		Absent
1994	IASP	Glossodynia and sore mouth	• *Definition*: Burning mouth syndrome (BMS) is a burning pain in the tongue or other oral mucous membrane persisting for at least four months and associated with normal oral mucosa and normal laboratory findings. • *Diagnostic criteria*: burning sensation in the tongue or other parts of the oral mucosa, usually bilateral and associated with dysgeusia, dry mouth and denture intolerance
2004	ICHD 2	BMS	• A. Pain in the mouth present daily and persisting for most of the day. • B. Oral mucosa is of normal appearance. • C. Local and systemic diseases have been excluded.
2013	ICHD 3	BMS	• A. Oral pain fulfilling criteria B and C. • B. Recurring daily for >2 hours per day for >3 months. • C. Pain has both of the following characteristics: ○ Burning quality. ○ Felt superficially in the oral mucosa. • D. Oral mucosa is of normal appearance and clinical examination including sensory testing is normal. • E. Not better accounted for by another ICHD-3 diagnosis.

ICHD, International Classification of Headache Disorders; IASP, International Association for the Study of Pain.

Overall, (i) relatively few studies have examined both cephalic and extracephalic somatosensory sensitivity in BMS patients, (ii) available studies have serious methodological limitations, and (ii) teams have neither replicated their own findings nor confirmed findings from other teams.

Studies selected in this review included more than 80% of females, around 60 years-old. This fits the known epidemiological characteristics of BMS patients [53,54]. Sample sizes in almost all included studies were rather small: the average numbers of subjects per study were 20 (range 8–40) and 23 (range 8–41) in the BMS and control groups, respectively. According to our estimate, detecting a difference of 2°C between healthy subjects and patients with a standard deviation of 3, a power of 80% and an alpha risk of 5%, requires at least 36 subjects per group. Thus, all studies had small or extremely small sample sizes, precluding firm conclusions. In addition, information on age and sex was sometimes not available [33,35,36] and, in one study [33], the number of subjects varied according to the section and stimulation modality. Of note, BMS patients and control subjects were often not properly sex-matched [37,38]. As orofacial sensitivity is influenced by sex, this is a major confounding factor [55]. Thus, the observed differences in some studies could be attributed to sex difference between patients and controls. Moreover, variables such as psychiatric condition, emotional, cognitive or dietary behavior (excessive consumption or avoidance of sugary, acidic, spicy…), that might influence pain sensitivity are rarely considered. Finally, stimulation parameters vary from one study to the other, making direct comparisons difficult. Thus, only 4 QST studies have no major methodological problems and could be considered as acceptable [32,33,35,39]. Nevertheless, even the results from these very studies are variable and sometimes contradictory.

### Is BMS a distinct entity or part of a global pain disorder?

Although there are many pathophysiological hypotheses, the mechanisms of BMS remain enigmatic. For instance, BMS was proposed to belong to ‘‘dynias”, a group of chronic focal pain syndromes localized to the orocervical and urogenital regions, including vulvodynia [11]. But, whereas TMD is frequently associated with vulvodynia [14,56], we found very few BMS-dynia co-occurrence: it was reported in only 7 patients within the retrieved studies [16,30]. Thus, whether BMS is a dynia remains an open question. Such an association, observed in very few patients, might be due to chance rather than to some putative similar contributing factors.

Central sensitization (CS) has also been suggested to underlie syndromes for which no specific organic cause can be found [15]. Disorders related to CS have been referred to as ‘‘central sensitivity syndrome (CSS)” [15]. They include fibromyalgia, irritable bowel syndrome, vulvodynia, tension headaches, migraine and TMD. BMS has also been proposed to belong to CSS [15]. However, a CSS disease should fulfill three criteria: 1) evidence for CS, 2) association with another CSS disease, based on studies with matched control groups or well-designed age and gender matched control populations, and, 3) absence of any underlying disease that could induce CS. Clearly, BMS does not meet all these criteria. Indeed, BMS patients frequently exhibit intraoral sensory deficits rather than allodynia or hyperalgesia [1–4] Moreover, there is no evidence for abnormal cutaneous cephalic and extracephalic sensitivity [31–39]. Finally, the association of BMS with other pain syndromes is not higher than that reported in the age-matched general population [43–50]. Altogether, these findings suggest that BMS is a distinct disease entity localized intraorally that depends on specific mechanism, probably at the peripheral trigeminal level. The involvement of the trigeminal system in the pathogenesis of BMS is supported by several recent findings [1–4]. First, psychophysical studies have repeatedly reported changes in the somatosensory sensitivity of the tongue [1–4]. Second, immunohistochemical studies have demonstrated a significant loss of epithelial and subepithelial nerve fibers together with an increased expression of NGF, TRPV1 ion channels as well as CB2 and P2X3 receptors in the tongue mucosa of BMS patients [1–4]. Third, BMS is associated with some changes in trigeminal reflexes [1–4]. Finally, the success of topical treatments in some forms of the disease provides further evidence for the trigeminal system involvement [1–4].

## Conclusions

The present systematic review reveals that (i) there is no evidence for an association between BMS and other pain symptoms and (ii) BMS patients do not display clear somatosensory patterns. The lack of co-occurring pain symptom with BMS suggests that this chronic pain syndrome depends on specific mechanisms, probably at the trigeminal level. By challenging several previous conclusions, this review clarifies the current state of knowledge about BMS. It strengthens the need for well-designed clinical studies to decipher its mechanism, using the most recent criteria to define BMS, appropriate sample sizes and age- and sex-matched controls.

## Supporting Information

S1 Fig PRISMA Checklist. (DOC)
